# Interspecific differences of stridulatory signals in three species of bark beetles from the genus *Polygraphus* Er. (Coleoptera: Curculionidae, Scolytinae) inhabiting the island of Sakhalin

**DOI:** 10.7717/peerj.8281

**Published:** 2020-01-02

**Authors:** Ivan Andreevich Kerchev

**Affiliations:** 1 Institute of Monitoring of Climatic and Ecological Systems of the Siberian Branch of Russian Academy of Sciences, Tomsk, Russian Federation; 2 Department of Forestry and Landscape Construction, National Research Tomsk State University, Tomsk, Russian Federation

**Keywords:** Bark beetle, Acoustic signals, Stridulation, Interspecific signals, Chirp

## Abstract

Stridulatory signals are involved in conspecific interactions between bark beetles (Coleoptera: Curculionidae, Scolytinae). In this study, we compared the qualitative profiles of acoustic signals in three species from the genus *Polygraphus* Er. Sympatry can be periodically observed in two of them –*P*. *proximus* and *P*. *subopacus*. Sporadically they occur on the same plants. *P*. *nigrielytris* colonize distinctly different host plant species; however, on the island of Sakhalin it inhabits the same biotopes. The purpose of the study is to identify species-specific parameters and the extent of differences in stridulatory signals of these species. Airborne signals produced during the contact of males of the same species were experimentally recorded. Among tested parameters of stridulatory signals, as the most species-specific were noted: chirp duration, number of tooth-strikes per chirp, and intertooth-strike interval.

## Introduction

Airborne sounds and solid-borne vibrations are widely used by animals as communication signals (Dumortier, 1963; Greenfield, 2002; Cocroft & Rodriguez, 2005). According to one of the latest generalized assessments, vibrational signals by 92% of all described insect species for communication (Cocroft & Rodriguez, 2005). Numerous studies on this type of communication analyze variability of inter and intra species signals among grasshoppers, crickets, and cicadas (Gerhardt & Huber, 2002; Greenfield, 2002; Greenfield, 2016; Boulard, 2005; Heller, 2005; Henry, 2005; Hoikkala, 2005; Sueur, 2005; Stewart & Sandberg, 2005). A group of leading researchers investigating different aspects of the transmission and reception of vibration signals proposed a conception of new term “semiophysicals” (Mazzoni et al., 2018) for vibrational signals to underline their similarity, in terms of functions, with semiochemicals (Blum, 1996).

It should be noted that the mechanisms of sound production and reception are also widespread among Hymenoptera, Hemiptera, and Coleoptera, which remain poorly studied in this regard despite their predominant species diversity (Kojima, Ishikawa & Takanashi, 2012; Breidbach, 1986; Wessel, 2006). Insects that live both on the surface and inside plants are of particular interest since plants are good mediators of vibrational signals (Michelsen et al., 1982; McVean & Field, 1996).

Bark beetles (Coleoptera: Scolytinae) produce signals using stridulation—a method of producing sounds by rubbing a scraper-like structure “plectrum” against a special file, consisting of a series of ridges called the “pars striden” (Barr, 1969; Ryker & Rudinsky, 1976; Yturralde & Hofstetter, 2015). It has been suggested that the stridulation signals of *Ips pini* Say females may have potential for protection against predators (Lewis & Cane, 1990), but this hypothesis has not been confirmed in further studies (Sivalinghem, 2011). It is still not clear whether airborne or solid-borne signals are perceived by these insects (Fleming et al., 2013; Dobai et al., 2017).

Almost all Holarctic representatives of the genus *Polygraphus* Er. occur on Pinaceae (Krivolutskaya, 1996; Nobuchi, 1979; Wood & Bright, 1992)—for example, the fir bark beetle *P subopacus* is specific to *Picea* spp., and the four-eyed fir bark beetle *Polygraphus proximus* attacks almost exclusively *Abies* Mill. trees (Wood & Bright, 1992). However, there are exceptions; for example, *P*. *nigrielytris* can be found exclusively on Angiosperms, *Sorbus* L., and *Alnus* Mill. in particular (Krivolutskaya, 1996).

In this study, we conducted a quantitative analysis of acoustic signals of three species, and two of these, *P. proximus* and *P. subopacus*, can be sporadically found on the same host plant. The third species, *P*. *nigrielytris*, is distinctly different from the former two species according to host-plant specialization (Krivolutskaya, 1958). The purpose of the study was to reveal the variants and the degree of differences in stridulatory signals required for interspecific differentiation of bark beetles within the genus *Polygraphus*, which are allopatric and sympatric with regard to the host plant.

## Materials & Methods

### Collection and storage of insects

Imagoes of three tested bark beetle species were collected from the brood trees *P. proximus* on *Abies sahalinensis, P. subopacus* on *Larix gmelinii*, and *P*. *nigrielytris* on *Sorbus commixta* in May 2018 on the island of Sakhalin in the territory of Krasnogorsky State Nature Reserve (48°29′22,2″N, 142°1′497″W). The field collection carried out in accordance with the institution field study approval (number 52) and the field collection protocol approved by the Sakhalin Forest Ministry (project OΓ378-42B). Species and sexual identification were performed based on morphological characteristics (Stark, 1952; Krivolutskaya, 1958). Unmated insects were placed individually in separate marked 5-ml glass tubes with a moistened cotton plug and were stored for one day at 4 °C before the recording procedure was performed.

### Morphological measurements

**Figure 1:**
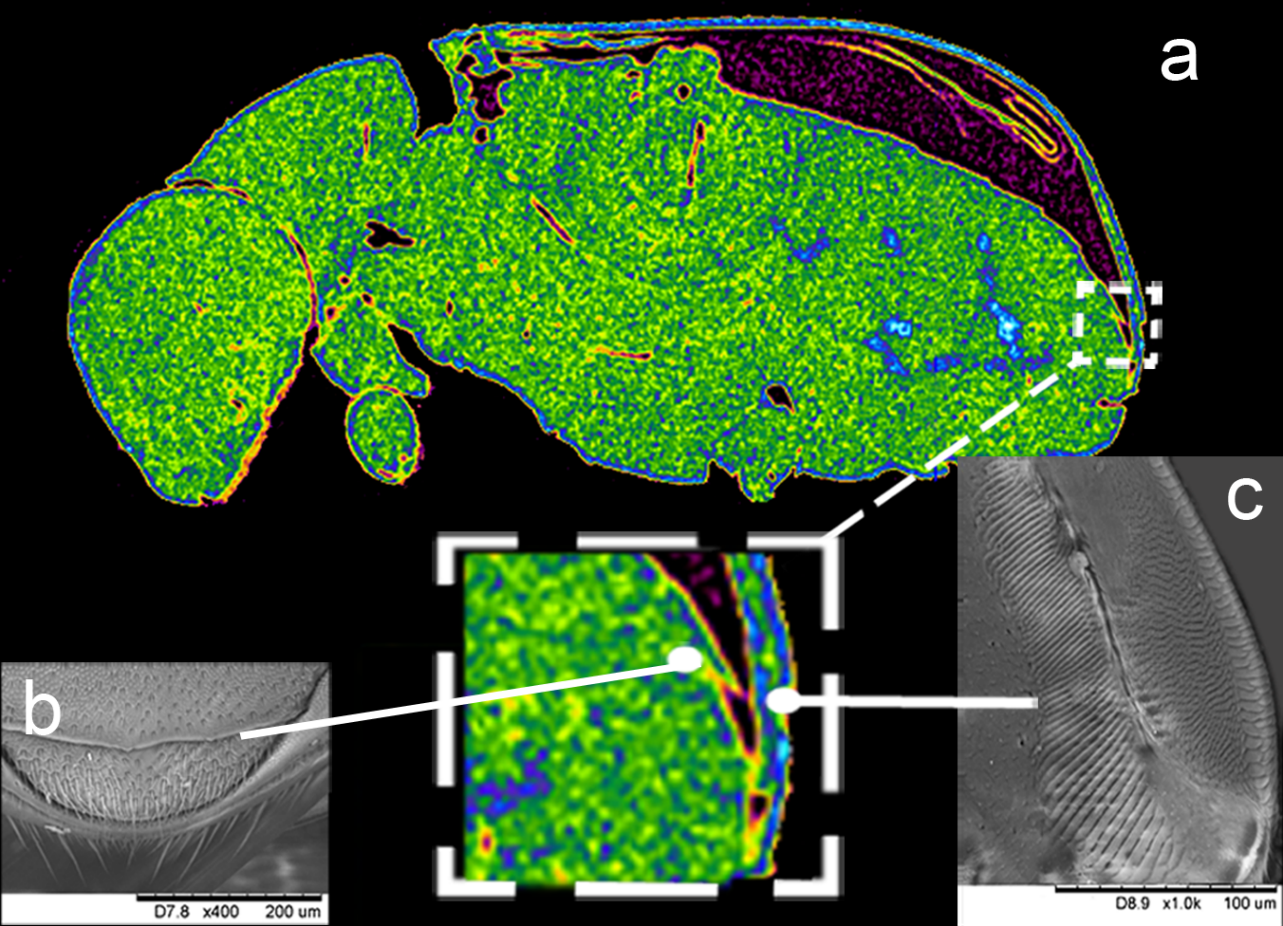
General view of elytro-tergal stridulatory apparatus morphology. (A) Longitudinal section of the imago prepared using an X-ray microtomography device; image of the elytron-tergite stridulatory apparatus of males; (B) detailed structure of the plectrum; (C) pars stridens structure.

**Figure 2:**
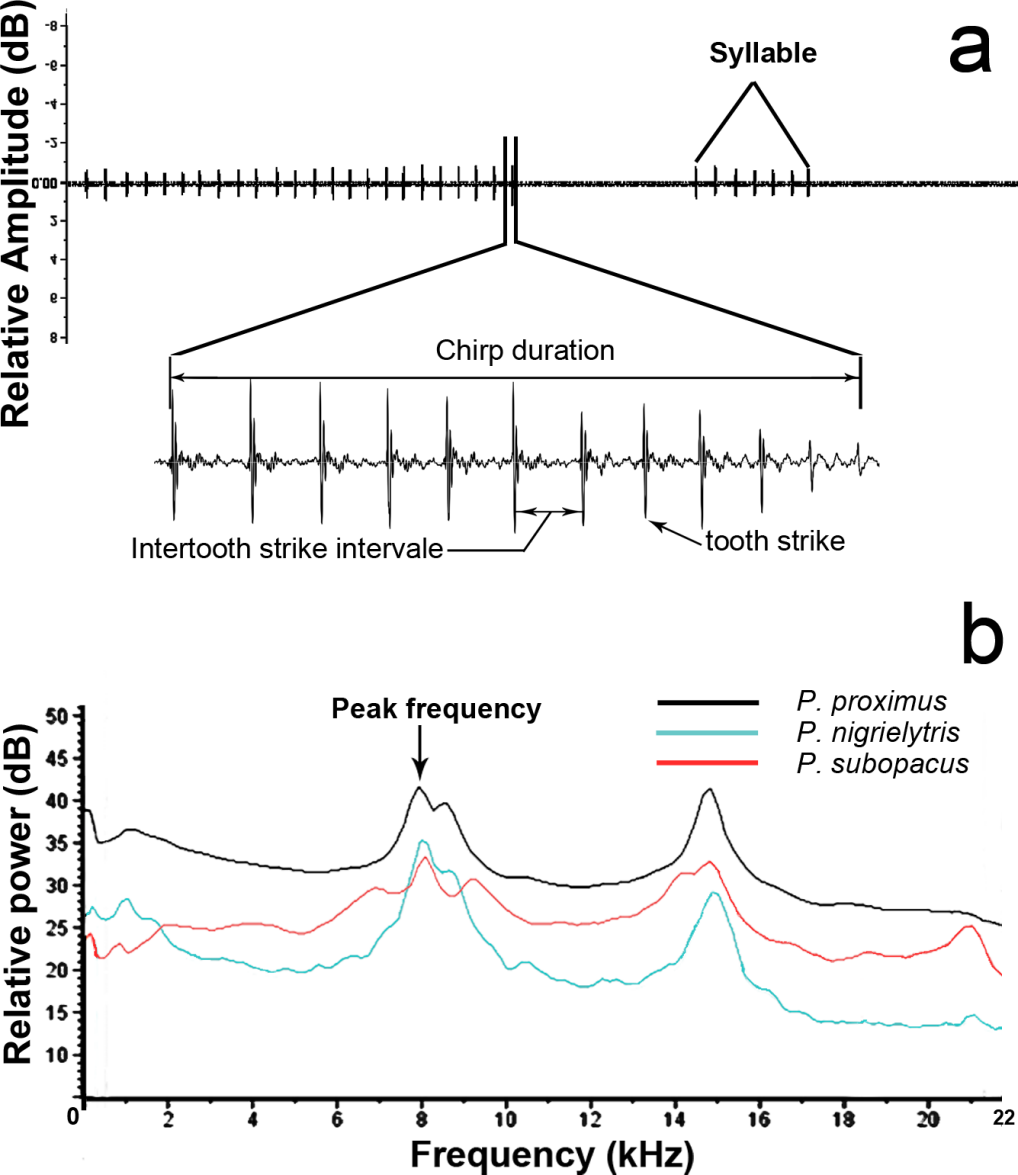
Descriptions of temporal and frequency-amplitude parameters measured in male stridulatory signals. (A) Descriptions of temporal parameters; (B) frequency-amplitude parameters of male signals.

An image of a longitudinal section of the imago was generated using an X-ray microtomography device (XWT 160-TC, X-RAY WorX; Garbsen, Germany) at Tomsk Polytechnic University (Fig. 1A). The images of the elytron-tergite stridulatory apparatus of males were prepared using a Tabletop Hitachi (Tokyo, Japan) 3000 TM scanning electron microscope (Figs. 2B, 2C) at Tomsk State University. Morphological characteristics such as pars stridens and number of ridges (Yturralde & Hofstetter, 2015; Kerchev, 2019) were measured using Levenhuk ToupView software (release date—10/15/2015; Levenhuk LabZZ, Tampa, United States).

### Design

Male–male interactions were recorded inside the arena (diameter of one cm), and a tube with a microphone was installed inside (Kerchev, 2019). No individual was used more than once for a given trial. For each species, recordings of 30 pairs of beetles were collected. For further analysis, we selected 60 (20 per species) files containing clear distinguishable signals and fewer noises.

Audio recording was performed using a Behringer condenser microphone (Willich-Münchheide II, Germany) (model: ECM 8000; 15–20,000 Hz) and a Zoom R16 digital recorder (Tokyo, Japan); frequency range: 20 Hz–44.1 kHz; sampling rate: 24-bit). The recorded signals, which lasted for 10–15 min, were saved using the WAV file format. The recording procedure was carried out in the Krasnogorsk forestry office (Krasnogorsk, Sakhalin region) in a cylindrical semi-anechoic chamber (diameter = 110 cm, *h* = 31 cm) covered with an echo-absorbing coating (2-cm wave), and the microphone was positioned inside the arena (glass tube diameter of one cm). During the recording procedure, the membrane of the microphone was located at a distance of 1.5 cm directly above the beetles (Kerchev, 2019). The recorded signals were analyzed in the Laboratory of Monitoring of Forest Ecosystems, IMCES SB RAS (Tomsk).

### Terminology and measurements

For each recording, several characteristics were analyzed—that is, syllable duration, number of chirps per syllable, chirp rate, chirp duration, interchirp interval, number of tooth-strikes per chirp, and the intertooth-strikes interval—according to the terminology proposed in previous studies (Pureswaran et al., 2016; Kerchev, 2019). Individual chirps were identified with the band-limited energy detector using Raven Pro 1.5 (Cornell Lab of Ornithology; Ithaca, New York) (Charif, Waack & Strickman, 2010). Peak frequencies and amplitudes were measured using the spectrogram slice view (Fig. 2).

The background noise was reduced and the signal was amplified using default parameters in the adaptive filtering option of Raven Pro 1.5 (Charif, Waack & Strickman, 2010). Spectra were also produced using a 512-point Fast Fourier Transform Hamming window. In the analysis each syllable was defined as a series of repeated chirps separated by an interval of silence. As a minimum interval for separating syllables was used a pause equal to three average intervals between adjacent chirps individually calculated for each record (Kerchev, 2019).

### Statistical analysis

The listed characteristics in each record were measured at 5 points calculated with the RANDBETWEEN function in Microsoft Excel. For analysis, we selected the chirps closest to the generated random points and all of the syllables throughout the whole record. Considering that we operated with relative values of the amplitude and energy of the signals, these values are not included in the statistical tests. Other parameters were compared using the Kruskal–Wallis test; for statistically significant differences, multiple comparisons were performed using a Bonferroni–Dunn post hoc test. All of the statistical analyses were conducted using Statistica 8.0 (StatSoft Inc.; Tulsa, OK, USA).

## Results

For *P*. *proximus* and *P*. *subopacus* males selected for recording, the accuracy of sexual separation was 100%. Verification of sexual identification of *P. nigrielytris* was carried out after recording the signals and fixation in alcohol and was found to be 65% due to a less pronounced sexual dimorphism that contrasted the other two tested species.

We obtained sound recordings of the male stridulations of the three tested species. We did not attempt to record any kind of female song due to the absence of any stridulatory apparatus on the elytra, as noted previously (Kerchev, 2019) for *P. nigrielytris* using collection materials from 2015. It has been established that *P*. *nigrielytris* males possess the largest areas of pars stridens with the greatest number of ridges (Table 1).

**Table 1:** Comparison of the morphological characteristics of the stridulator apparatus and average values of the parameters of competitive signals in males of Polygraphus proximus, *P. nigrielytris* and *P. subopacus* (Mean ± SD).

**Parameter**	***P. proximus***	***P. nigrielytris***	***P. subopacus***
Stridulating sex	male	male	male
Length of beetle (mm)	2.5 ± 0.2	2.7 ± 0.1	1.9 ± 0.1
Length of pars stridens left/right (µm)	156/193	198/213	148/152
Number of rows in pars stridens	55/53	71/76	65/66
Syllable duration (s)	14.4 ± 8.4	4.4 ± 2.7	8.4 ± 6.1
Number of chirps /syllable	14.8 ± 18.1	19.1 ± 13.3	26.3 ± 21.4
Chirps rate (chirps/s)	5.1 ± 0.9	4.4 ± 0.9	3.9 ± 0.9
Chirps duration (s)	0.025 ± 0.008	0.020 ± 0.007	0.042 ± 0.001
Interchirp interval (s)	0.17 ± 0.4	0.23 ± 0.04	0.26 ± 0.05
Number of tooth-strikes	13.4 ± 4.0	10.5 ± 3.2	7.5 ± 2.2
Intertooth-strikes interval (s)	0.002 ± 0.0006	0.001 ± 0.0005	0.004 ± 0.001
Peak frequency (kHz)	7960.7 ± 42.27	8017,95 ± 65,21	8715 ± 2113
Relative power of signal (dB) at 1.5 cm distance	41.7 ± 5.3	34.2 ± 7.0	33.0 ± 5.3

The highest density of ridges in pars stridens was noted for *P*. *subopacus* (Table 1). Significant differences were found in the syllable duration (H (2, 60) = 23.8; *p* < 0.001) and the chirp rate (H (2, 60) = 10.7; *p* < 0.005) between species (Table 2). Nevertheless, the interchirp interval duration did not show statistically significant differences between diferent species signal.

**Table 2:** Results of pairwise comparison of parameters of competitive signals in males of Polygraphus proximus, *P. nigrielytris* and *P. subopacus*.

**Parameter**	**Species**	*P. subopacus*	*P. nigrielytris*
Syllable duration (s)	*P. proximus*	2.6	4.9^**^
	*P. subopacus*		2.3
Chirp rate (chirp/s)	*P. proximus*	3.2^*^	1.3
	*P. subopacus*		2.1
Chirp duration (s)	*P. proximus*	3.6^**^	3.1^*^
	*P. subopacus*		3.6^**^
Tooth strikes /chirp	*P. proximus*	6.2^**^	3.1^*^
	*P. subopacus*		3.1^*^
Intertooth strike interval (s)	*P. proximus*	3.2^*^	0.3
	*P. subopacus*		3.4^**^

**Notes.**

*Z*-values in cells.

* *p* < 0.05.

** *p* < 0.001 with Bonferroni correction.

Significant differences were identified between the signals of the tested species for the chirp duration parameter (H (2, 60) = 15.5; *p* < 0.001). In this parameter, *P. proximus* and P*. nigrielytris* are more distinguishable from *P. subopacus* then between each other (Table 2). The number of tooth strikes/chirp (H (2, 60) = 38.8; *p* < 0.001) showed the highest species specificity. In the pairwise comparison of this signal parameter, statistically significant differences were found for all pairs of species that were compared (Table 2). The interval duration between tooth strikes showed significant differences except for *P. nigrielytris* and *P. subopacus*.

The energy was found to be mostly concentrated between 2,000 and 22,000 Hz, which is within the range of human hearing, and the two most noticeable peaks were at approximately 8 and 14 kHz (Fig. 2). The average values of the main peak of energy are shown in Table 1.

## Discussion

Insects were collected at the beginning of the spring dispersal of the four-eyed fir bark beetle. Similar to timing in the secondary range (Kerchev, 2014), this species has the earliest spring flight starting period among the bark beetles on Sakhalin (Krivolutskaya, 1958). At the moment of collection, the majority of the beetles of this species was mature, and they had already left the brood tree or were ready for dispersal. In the galleries of *P. subopacus* and *P. nigrielytris* under the bark of brood trees, prepupae pupae and young beetles with light exoskeletons were mainly observed. Mature adults were collected from well-lit and heated areas only for recording. Thus, in addition to host specificity, phenological isolation can be considered as one of the factors of interspecific isolation of the test species.

Behavioral differences between species can be identified through differences in mating systems. *P. subopacus* is the only harem-poligynous among the three species that were compared. The sex ratio in its nests is about 2–5 females per male (Stark, 1952), and the mating system of the other two species is monogynous. The sexual behavior of *P. proximus* was previously discussed (Kerchev, 2014), whereas data on the characteristics of the sexual behavior of *P. nigrielytris* are given for the first time. During insect collection, only a single pair of parent beetles was always found in nests inhabited by beetles in spite of the fact that the number of egg galleries was 1–4.

The morphological characteristics of the stridulatory apparatus of the *P. proximus* were reported earlier (Sasakawa & Yoshiyasu, 1983), after which they were specified and supplemented (Kerchev, 2015). The presence of the stridulatory apparatus in *P. subopacus* was identified for the first time more than a hundred years ago, but the morphology description was not provided (Wichmann, 1912; Lyal & King, 1996). For *P. nigrielytris*, this study indicates the presence of stridulation and the morphological features of the structures involved in the sound production for the first time (Table 1). In general, the species of the genus *Polygraphus Er.* are similar in terms of the morphology of their stridulatory apparatus, but they are different in terms of morphometric features. An intraspecific comparison shows variations in the area and the number of ridges in pars stridens (Kerchev, 2015), and the density of ridges per unit length of the area can be noted as a more stable feature.

**Figure 3:**
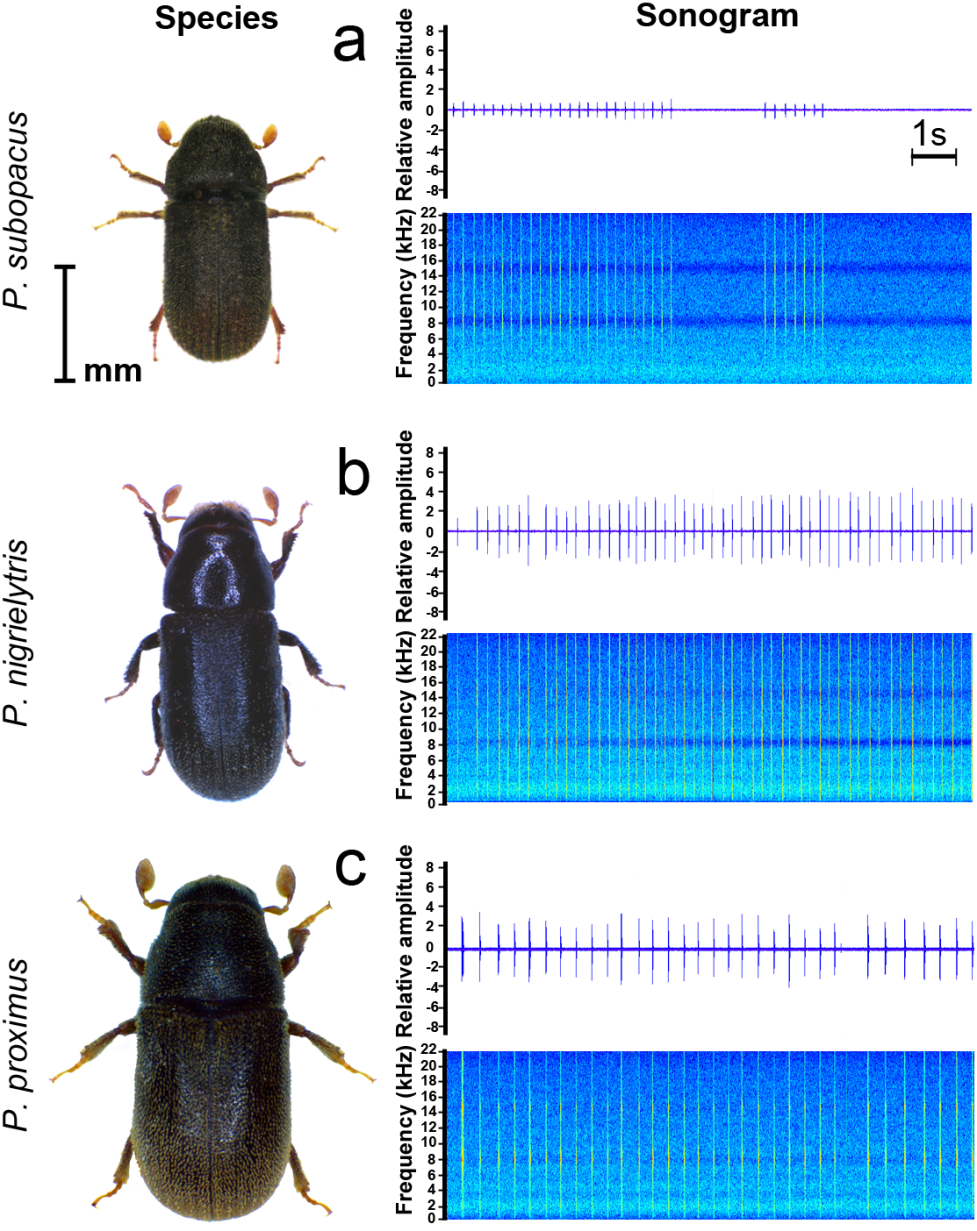
Adults of tested species with fragments of their signal samples: (A) *Polygraphus subopacus* (B) *P. nigrielytris*, (C) *P. proximus*.

Like many other insects, bark beetles are physically limited in terms of producing sounds due to their small size (Bennet-Clark, 1998). The studied species exhibit noticeable differences in the relative amplitude of signals, which may be due to the size of the insects (Table 1), especially in *P. subopacus* (Fig. 3). Among cicadas and crickets, the smallest species produce signals with the highest frequency compared with those produced by larger species. A similar negative correlation between body size and frequency parameters was noted earlier for bark beetles of the genus *Dendroctonus* Er. (Yturralde & Hofstetter, 2015). This study revealed significant differences between the stridulatory signals of the studied species in five of the seven temporal parameters. No differences were found in parameters such as number of chirps per syllable and duration of intervals between them.

The signal parameters showed the highest specificity, starting with the level of chirp, which is primarily due to the physiological characteristics of the species and the morphology of the microstructures of their stridulatory apparatus (Pureswaran et al., 2016; Kerchev, 2019).

It was experimentally found that a rather short fragment of the signal consisting of 14 pulses repeated at least once per minute is sufficient for females of the bush-cricket *Metrioptera roeselii* Hagen. to recognize an intraspecific attractive signal (Zhantiev & Korsunovskaya, 2014). In the case of competitive interaction between *P. proximus* males, the contact lasts more than one minute only during a fight for a female boring into the bark. In other cases, the male stays at the entrance to the gallery occupied by a formed family for not more than several seconds (Kerchev, 2019). Consequently, the territorial signal must have the characteristics that would allow it to be recognized in a short period, and the chirp as a signal unit has all of the necessary characteristics. Under experimental conditions, continuous stridulation may be caused by limited abilities to escape contact between individuals inside the arena.

A number of different ethological considerations that have already been mentioned support interspecific isolation and communication features. Species-specificity of stridulatory signals may be an additional parameter that performs the same role for individuals that have started to populate a tree beyond the main dispersal flight period. During male interactions of different species, the signal receiver may not regard it as a repellent. A clear repellent reaction during conspecific interaction can indicate a crucial role of these signals in reduction of intraspecific competition.

To date, a number of research papers (Mankin et al., 2008; Potamitis, Ganchev & Fakotakis, 2008; Schofield & Chesmore, 2010) are devoted to the use of species-specific insect signals for species identification. The possibility of identification of alien species and monitoring of their populations based on detection of their species-specific signals is of particular interest in this regard. Among the parameters tested, the most relevant parameters are syllable duration, the interval between syllables, the number of syllables per unit of time, and the relative amplitude of signals.

## Conclusions

This study showed significant differences in temporal parameters of signals both between species occupying the same ecological niche and with congener which interspecific isolation is already ensured by allopatry.

The species-specificity of stridulatory signals may be an additional parameter for reproductive isolation of species that occasionally occur on the same tree species. Reception and reaction to this type of signals may be present both at the interspecific level of interactions and during intraspecific contacts only. To verify the possibility of interspecific communication at the level of one genus, it is necessary to conduct playback experiments by recording responses to alternating con- and interspecific signals. Biologically, signals produced by males of one species may reduce intraspecific competition at high population densities.

Of particular interest is the possibility of using species-specific characteristics of acoustic signals of bark beetles for identification and detection of alien species. Among the tested parameters of stridulation signals, the following specific characteristics can be distinguished for the genus *Polygraphus*: chirp duration, number of tooth-strikes per chirp, and intertooth-strike interval. Despite the limitations associated with the analysis, which was performed on the average of acoustic data and sometimes included signals of both males, this method can be used universally to compile libraries of species-specific signals in order to further develop methods for detection and species identification of bark and wood-boring pests.

## Additional Information and Declarations

### Field Study Permissions

The following information was supplied relating to field study approvals (i.e., approving body and any reference numbers): Field collection were approved by Sakhalin forest ministry (project G378-42V; institution field study approval number: 52).

### Data Availability

The following information was supplied regarding data availability: The raw data is available at Figshare: Kerchev, Ivan (2019): Polygraphus stridulatory signals. figshare. Media. 10.6084/m9.figshare.8341334.v1.
